# Relationship between hemagglutinin stability and influenza virus persistence after exposure to low pH or supraphysiological heating

**DOI:** 10.1371/journal.ppat.1009910

**Published:** 2021-09-03

**Authors:** Guohua Yang, Chet R. Ojha, Charles J. Russell

**Affiliations:** 1 Department of Infectious Diseases, St. Jude Children’s Research Hospital, Memphis, Tennessee, United States of America; 2 Department of Microbiology, Immunology & Biochemistry, College of Medicine, The University of Tennessee Health Science Center, Memphis, Tennessee, United States of America; Emory University School of Medicine, UNITED STATES

## Abstract

The hemagglutinin (HA) surface glycoprotein is triggered by endosomal low pH to cause membrane fusion during influenza A virus (IAV) entry yet must remain sufficiently stable to avoid premature activation during virion transit between cells and hosts. HA activation pH and/or virion inactivation pH values less than pH 5.6 are thought to be required for IAV airborne transmissibility and human pandemic potential. To enable higher-throughput screening of emerging IAV strains for “humanized” stability, we developed a luciferase reporter assay that measures the threshold pH at which IAVs are inactivated. The reporter assay yielded results similar to TCID50 assay yet required one-fourth the time and one-tenth the virus. For four A/TN/09 (H1N1) HA mutants and 73 IAVs of varying subtype, virion inactivation pH was compared to HA activation pH and the rate of inactivation during 55°C heating. HA stability values correlated highly with virion acid and thermal stability values for isogenic viruses containing HA point mutations. HA stability also correlated with virion acid stability for human isolates but did not correlate with thermal stability at 55°C, raising doubt in the use of supraphysiological heating assays. Some animal isolates had virion inactivation pH values lower than HA activation pH, suggesting factors beyond HA stability can modulate virion stability. The coupling of HA activation pH and virion inactivation pH, and at a value below 5.6, was associated with human adaptation. This suggests that both virologic properties should be considered in risk assessment algorithms for pandemic potential.

## Author summary

Many influenza viruses circulate among wild aquatic birds, sporadically infecting domestic animals and humans. Avian influenza viruses need to “humanize” several properties before efficiently replicating in mammals and transmitting between humans. To triage emerging zoonotic viruses for pandemic likelihood and pathogenic impact in humans, the Centers for Disease Control and Prevention (CDC) and World Health Organization (WHO) use risk assessment algorithms that consider viral properties, human population attributes, and non-human virus ecology. While receptor-binding specificity of the HA protein is the only molecular property explicitly considered, recent studies have shown the HA protein needs to become stabilized to enable human adaptation and airborne transmission in ferret experimental models. In this work, we developed a high-throughput assay to measure the HA stability of surveillance isolates by monitoring the pH at which these viruses become inactivated. Our results showed HA activation pH and virion inactivation pH are similar for human viruses but differ in some animal viruses. Knowing that these two values, previously considered equivalent, can differ substantially will spur researchers to determine the relative importance of each for humanizing emerging influenza viruses so that pre-pandemic preparations can better target viruses of greatest risk to humans.

## Introduction

Genetically diverse influenza A viruses (IAVs) circulate in wild aquatic birds, wild animals (e.g., turkeys, whales, seals, mink), domestic animals (e.g., poultry, horses, canines, swine), humans, and most likely bats [1]. The ability of an IAV from one species to attain sustained transmission in a different species depends on a variety of viral genetic and host factors [2, 3]. During viral entry, hemagglutinin (HA) surface glycoprotein trimers bind terminal sialic acid (SA) moieties expressed on the surfaces of host cells [4, 5]. Avian IAVs preferentially bind glycans with SA α-2,3-linked to its subterminal galactose, while those adapted to humans and ferrets contain mutations in the receptor-binding pocket that allow engagement of glycans with α-2,6-linked SA [6–8]. IAV virions and IAV-infected cells contain terminal glycans, and the neuraminidase (NA) surface glycoprotein cleaves terminal SA moieties from these glycans to prevent virus aggregation and superinfection [9]. Airborne transmission between humans has been correlated with a functional balance between HA affinity for SA-containing receptors and NA enzymatic activity to cleave SA [10–12]. In addition to the HA and NA surface glycoproteins, viral internal genes and their interactions with host cell proteins also regulate interspecies adaptation of influenza viruses.

Viral RNA binding by retinoic acid-inducible gene I protein (RIG-I) initiates a cascade that results in antiviral type I and type III interferon expression [13]. The polymerase basic protein 2 (PB2) proteins of avian IAVs have a glutamic residue 627, and human-adapted PB2 proteins may contain E627K mutations that increase polymerase activity and virulence in humans and other mammals [14, 15]. Other polymerase variations such as PB2-D701N have also been associated with human adaptation by IAVs [16]. Identification and surveillance of viral characteristics needed for IAV adaptation to humans is the critical first step in pre-pandemic efforts to (a) assess circulating strains most likely to become humanized and cause impactful disease and (b) prepare emergency countermeasures such as culling infected animals, selecting vaccine seed strains, and preparing diagnostic reagents.

HA stability, or the ability of the surface glycoprotein to resist structural changes induced by mild acid or heating, has recently been shown to be necessary for the adaptation of IAVs to humans and ferrets (reviewed in [17]). After binding SA-containing receptors during entry, IAVs are internalized into endosomes [18, 19], where they are exposed to sequentially lower pH [20]. Mature HA proteins (i.e., those cleaved into HA1/HA2 complexes) are trapped in a metastable conformation that is triggered by low pH in endosomes to undergo irreversible structural changes that cause membrane fusion [21–24]. Threshold pH values for IAVs range approximately between pH 4.8 and 6.2 with human-adapted IAVs trending toward the lower end (usually 4.8–5.5) [17, 25, 26]. In tandem with a preference for HA binding to α-2,6-linked SA, a relatively stable HA (activation pH of 5.6 or lower) has been shown to be necessary for airborne transmission of avian, swine, and human IAVs between ferrets [27–34]. Activation pH values of HA proteins from several influenza viruses isolated during the 20^th^ Century have been reported to be approximately 5.0–5.2. These include A/Puerto Rico/8/1934 (H1N1), A/WSN/1933 (H1N1), A/Japan/305/1957 (H2N2), A/Aichi/2/1968 (H3N2), and A/Victoria/3/1975 (H3N2) [26]. Early 2009 pandemic H1N1 (pH1N1) isolates had HA activation pH values of approximately 5.5 [26, 34], and adaptation of pH1N1 to humans resulted in adaptive mutations that decreased the HA activation pH [35–37]. In an experimental study, a pH1N1 loss-of-function mutant (HA1-Y17H) with an elevated HA activation pH (6.0) regained airborne transmissibility in ferrets by two adaptive mutations (HA1-H17Y reversion and HA2-R106K) that decreased the HA activation pH to 5.3, mirroring the evolution of pH1N1 in humans [34].

Several non-exclusive mechanisms have been identified by which HA stability helps determine the host range of IAVs. A stabilized HA protein, or one with a lower activation pH value, is expected to prolong environmental stability and may, thereby, reduce virion inactivation in mildly acidic extracellular environments [38–40]. HA stabilization has been shown to promote pH1N1 infectivity after expulsion from infected ferrets [41]. Upon infection, the upper respiratory tracts of humans are mildly acidic [42, 43], so a stabilized HA might enhance intra-host spread of infection by avoiding extracellular inactivation of virions [17]. Inside cells, intermediate HA stability may be preferred [44] by minimizing interferon responses that are triggered either in dendritic cells by high-pH HA proteins in early endosomes [40] or by interactions between low-pH HA proteins and IFITM2 and IFITM3 proteins in late endosomes [45]. Other unidentified mechanisms may also be operational and vary by host and tissue.

When evaluating the human pandemic potential of emerging IAVs, risk assessment tools used by the WHO and CDC consider HA receptor-binding specificity [46, 47]. Additional consideration of HA stability in these algorithms would most likely enhance their predictive abilities [2, 3, 17]. A key challenge to adding HA stability to routine surveillance efforts is that the property must be measured phenotypically and cannot be inferred from the HA gene sequence for several reasons. First, residues that regulate HA stability are positioned throughout the primary sequence and tertiary structure [48, 49]. Second, mutations to over 50 amino-acid residues have been found to alter HA stability (reviewed in [17, 50, 51]). Third, X-ray crystal structures show that stability-altering HA mutations often do not perturb the HA protein backbone [52–54]; therefore, computer modeling based on sequence would not be expected to yield accurate values for overall HA stability. Finally, the NA and M proteins can also modulate HA stability [53, 55–57].

Several assays can be used to measure HA stability, although each can be time-consuming, cumbersome, highly technical, or strain-dependent and, thus, may not ideally suited for high-throughput measurement of this property. The pH or temperature required to induce HA conformational changes occur can be measured by conformation-specific protease digestion followed by SDS-PAGE [58–60] or reactivity with conformation-specific antibodies [58, 61–63]. The pH or temperature required to trigger HA-mediated membrane fusion can also be measured for IAV virions, infected cells, or HA-transfected cells. Virion-based assays can measure membrane disruption by hemolysis [57, 64] or membrane fusion by fluorescence dequenching, fluorescence energy transfer (FRET), or total internal reflection fluorescence microscopy (TIRF) [65, 66]. Membrane fusion between infected or transfected cells expressing HA proteins can be measured by heterokaryon (syncytia) formation [58, 63, 67, 68], dye transfer [65, 69] or reporter-gene expression [66]. Finally, the pH or temperature at which virions become inactivated can be quantified through exposure of virus aliquots to gradients of acidity or heat and measuring residual infectivity by standard methods [25].

The term HA stability has been used to express a relative resistance to triggering HA conformational changes, HA-mediated membrane fusion, and virion inactivation. However, it has not yet been established if these properties correlate with each other for all IAV strains and, if not, which aspect (s) contribute to interspecies adaptation. For example, HA activation pH and virion inactivation pH values for the same viruses are often similar [32, 34] but can deviate in some cases [70, 71]. The overarching goal of this study was to examine the relationship between HA activation pH and the ability of influenza virions to resist inactivation after exposure to mild acidification or heating. To support this work, we developed first a luciferase reporter-gene assay to measure virion infectivity.

## Results

### Measurement of pH-dependent virus inactivation using Luc9.1 luciferase reporter cells

**Fig 1:**
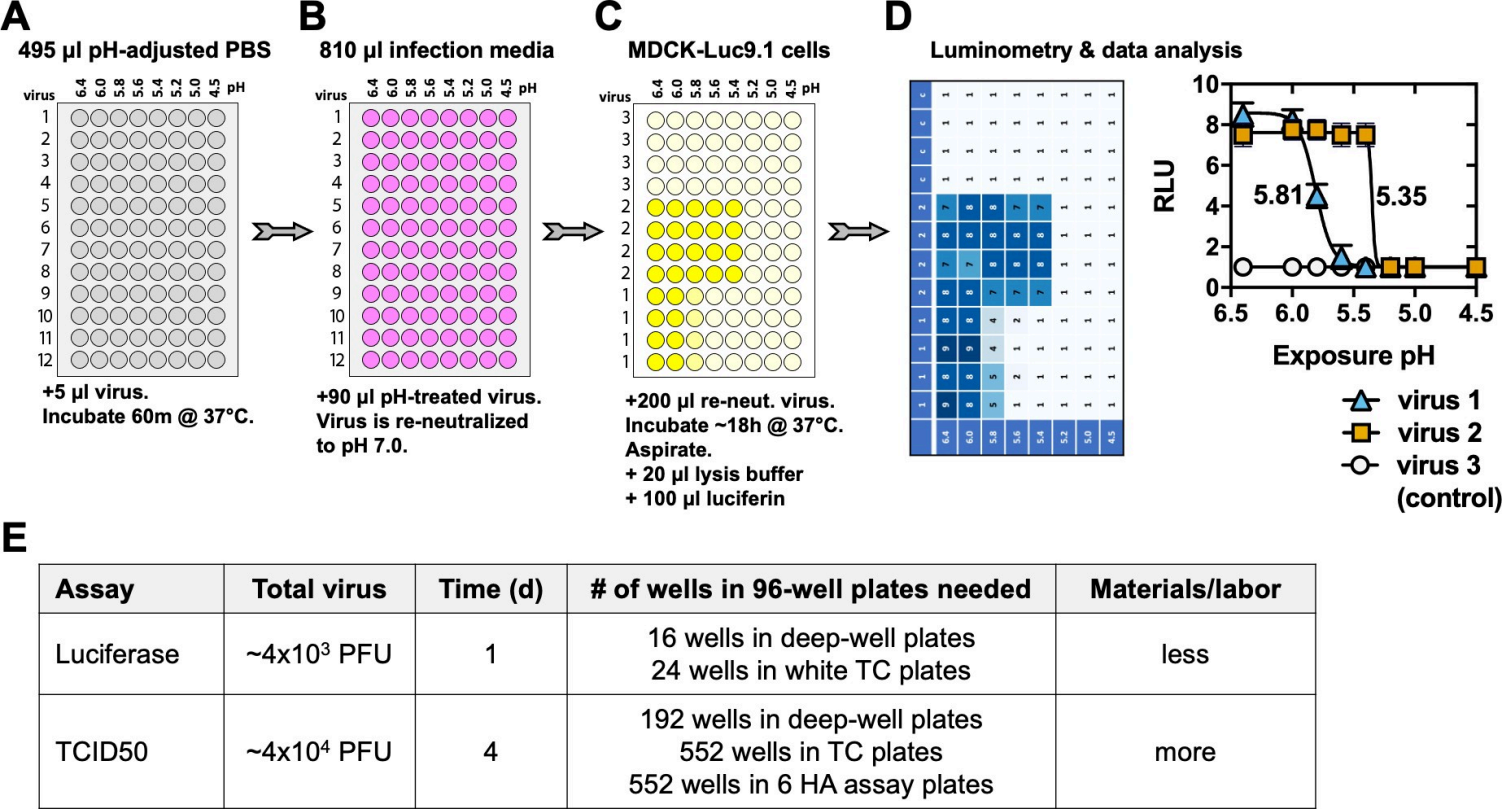
Schematic of luciferase reporter assay to measure influenza virus inactivation pH. (A) Acid treatment of samples. Into each well of a 96 deep-well plate, 495 μl of pH-adjusted PBS is added. 5-μl aliquots of twelve virus or control samples are added to eight wells each and incubated at 37°C for 1h. (B) Re-neutralization. A multichannel pipette is used to transfer 90 μl of sample from panel A into 810 μl infection media that has a pH of 7.0. (C) Infection of reporter cells. A multichannel pipette is used to transfer 200 μl re-neutralized virus to a white 96-well tissue culture (TC) dish containing MDCK-Luc9.1 cells. After 17-19h incubation at 37°C and 5% CO_2_, media is aspirated, 20 μl of lysis buffer is added, and 100 μl of diluted Renilla luciferase assay substrate is added. (D) Quantification of reporter gene expression and calculation of virus inactivation pH. Luminescence is measured using a plate-reader luminometer. Relative light units (RLUs) are output to csv files, which are then analyzed in GraphPad Prism 8 to calculate the point of inflection by nonlinear regression with a dose-response equation. In this example, samples include virus 1 (calculated pH 5.81), virus 2 (calculated pH 5.35), and virus 3 (control virus, used to estimate the baseline reading). RLU values were assigned for illustrative purposes only and are not real data. (E) Comparison of luciferase and TCID50 assays to measure virus inactivation pH. Total virus is an estimate of total PFUs needed for each assay. Time to conduct assay is after culturing of cells.

**Fig 2:**
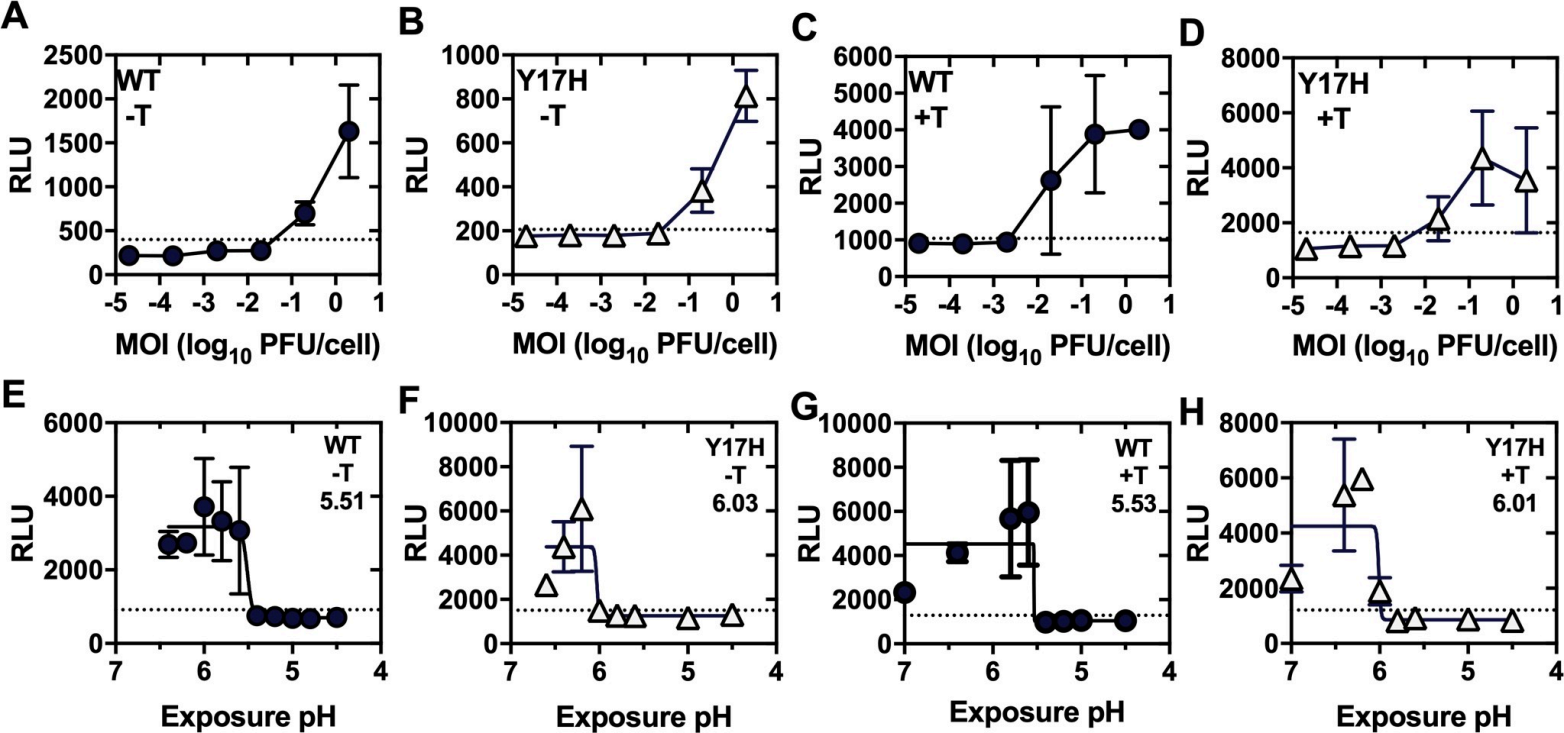
*Renilla* luciferase assay of residual infectivity after acid exposure using MDCK Luc9.1 reporter cells. MDCK Luc9.1 cells were inoculated with A/TN/09 WT (solid circles) or Y17H (open triangles) at MOI values ranging from 2x10^-5^ to 2 PFU/cell and were overlaid with media lacking TPCK-treated trypsin (A,B; -T) or containing it (C,D; +T). After 17 h, cell lysates were mixed with *Renilla* luciferase substrate and luminescence (relative light units, RLU) was measured using a 96-well plate luminometer. Virus aliquots in panels E-H were exposed to pH-adjusted PBS for 1h before inoculation into Luc9.1 reporter cells at an MOI of 2 (E,F) or 0.2 (G,H) PFU/cell. Data in panels E-H were fit by nonlinear regression (dose-response simulation) to calculate the midpoints of inactivation, or “inactivation pH”, which are listed on the panels. Dotted horizontal lines correspond to the lowest detectable residual infectivity after acid treatment (defined as 3 standard deviations above the mean of low-pH inactivated aliquots). Error bars represent standard deviation (n = 3). Reported data are representative of three independent experiments.

Well-established assays of influenza virus infectivity (i.e., TCID50, EID50, and plaque assay) take multiple days and require relatively large amounts of cultured cells or eggs, materials, and labor. To develop a more efficient assay to measure infectivity after exposure to buffers of varying pH, we used Luc9.1 reporter cells [72]. These influenza reporter cells are Madin-Darby canine kidney (MDCK) cells that constitutively express an influenza-like luciferase RNA reporter gene that is transcribed upon influenza infection since the luciferase amplicon is flanked by noncoding regions from the NP gene of A/WSN/33. In the present work, virus aliquots exposed to varying pH-adjusted PBS buffers were re-neutralized, inoculated into Luc9.1 cells, and assayed for luciferase enzymatic activity as a reporter for IAV infection (Fig 1). During assay development, A/Tennessee/1-560/2009 (H1N1) (A/TN/09) was used as a model virus [33, 34, 73]. To determine the minimum multiplicity of infection (MOI) required for luciferase reporter gene expression above background, Luc9.1 cells in 96-well plates were inoculated with 10-fold dilutions of wildtype (WT) virus or the mutant HA1-Y17H, which contains a destabilizing mutation in the HA2 stalk [33, 34, 73]. The MOI ranged from 2x10^-5^ to 2 PFU/cell. In the absence of exogenous trypsin, which is needed for virus amplification, an MOI of 0.2 PFU/cell or higher was required to yield average luciferase signals over background (Fig 2A and 2B). Addition of 1 μg/mL TPCK-treated trypsin, which allowed virus amplification, increased assay sensitivity approximately 10-fold to 0.02 PFU/cell (Fig 2C and 2D).

To measure the pH of virion inactivation, we diluted 5-μl aliquots of A/TN/09 WT or HA1-Y17H into 495 μl of pH-adjusted PBS at 0.2-unit increments for 1 h at 37°C, re-neutralized, inoculated Luc9.1 cells, and measured residual infectivity by luminescence after 17 h incubation. For WT virus, the midpoint of inactivation after exposure to acid (i.e., virion inactivation pH) was approximately 5.5, whether Luc9.1 cells were inoculated with 2 PFU/cell in the absence of TPCK-treated trypsin (Fig 2E) or 0.2 PFU/cell in the presence of TPCK-treated trypsin (Fig 2G). HA1-Y17H had an inactivation pH of approximately 6.0 under both conditions (Fig 2F and 2H). These inactivation pH values determined by luciferase assay were identical to those calculated from a previous study using TCID50 as a readout [34]. For subsequent luciferase assays of infectivity, we used 0.2-MOI infection in the presence of trypsin so that as little as 4,000 PFU virus would be needed per experiment compared to ~40,000 PFU needed for TCID50 assays. We used 0.2 pH-unit increments because larger spacing was not as accurate (S1 Fig).

### Inactivation pH values measured by luciferase assay and by TCID50

**Fig 3:**
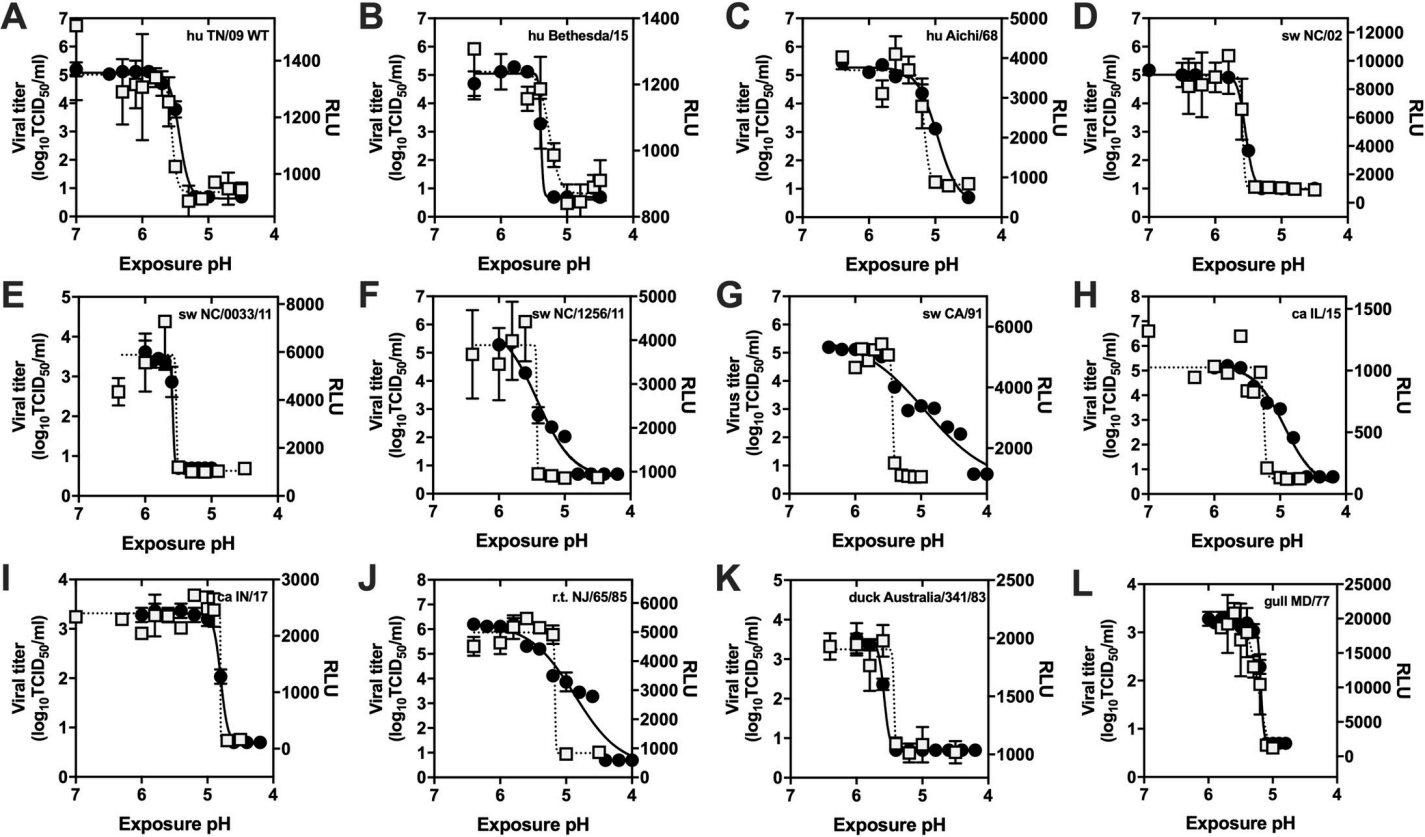
Virus inactivation measured by TCID50 and luciferase assays. TCID50 values (left y-axis, black circles) and luciferase activity (RLU, right y-axis, white squares) were measured as a function of exposure to the reported pH followed by re-neutralization. Right y-axes are scaled so that RLU maxima and minima are displayed at levels similar to those from TCID50 on the opposite axis. Error bars represent standard deviation (n = 3). Reported data are representative of two or more independent experiments. (A) rg-A/TN/1-560/2009 (H1N1). (B) A/Bethesda/55/2015 (H3N2). (C) A/Aichi/2/1968 (H3N2). (D) rg-A/swine/NC/18161/2002 (H1N1). (E) A/swine/North Carolina/0033/2011 (H3N2). (F) A/swine/North Carolina/1256/2011 (H3N2). (G) A/swine/California/T9001707/1991 (H1N1). (H) A/canine/Illinois/41915/2015 (H3N2). (I) rg-A/canine/Indiana/1177-17-1/2017 (PR8 internal genes) (H3N2). (J) A/ruddy turnstone/NJ/65/1985 (H7N3). (K) A/duck/Australia/341/1983 (H15N8). (L) A/gull/Maryland/704/1977 (H13N6).

**Fig 4:**
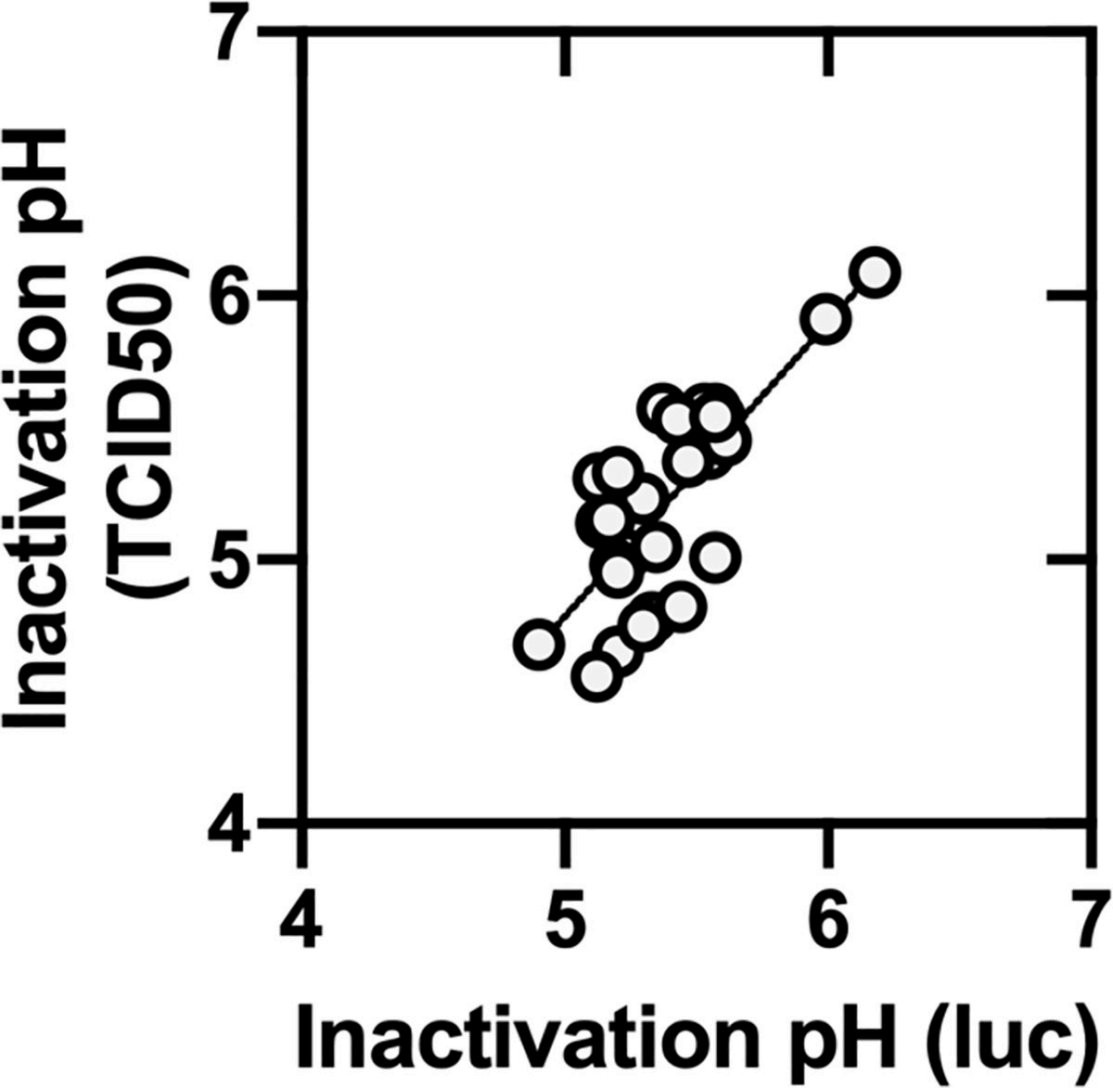
Comparison of virus inactivation values measured by TCID_50_ and luciferase assays. Inactivation pH values measured byTCID_50_ are reported as the calculated pH corresponding to a 2-log_10_ (100-fold) drop in infectivity measured by TCID_50_ assay. Inactivation pH values for luciferase assay are reported as the pH at the midpoint of the S-shaped luminescence curves. Both TCID_50_ and luciferase inactivation values were calculated using GraphPad Prism 8 using a nonlinear regression with log(agonist) vs. response and a four-paramter variable slope. The displayed line (R^2^ value of 0.604) was calculated by simple linear regression using GraphPad Prism 8.

Residual infectivity after exposure to acidic buffer has been measured by hemagglutination (HA) assay [25] and TCID50 [34]. To compare the newly developed luciferase assay to TCID50 for measuring virus inactivation pH, we performed both assays on 28 viruses (Table 1). These included seven human viruses (4 H1N1, 3 H3N2), eight swine (6 H1N1, 2 H3N2), three canine (3 H3N2), and ten avian (4 H1N1 and 1 each of H5N2, H5N8, H7N3, H13N6, H14N5, H16N3). All 28 viruses had a sharp drop in luminescence over a narrow pH range of approximately 0.1–0.2 units (Fig 3). Most viruses had similarly sharp decreases in virus infectivity measured by TCID50 (cf. Fig 3A–3E, 3I, 3K and 3L). Some isolates had relatively broad inactivation curves in the TCID50 assay, but not in the luciferase assay (cf. Fig 3F–3H and 3J). This suggests TCID50 may be more sensitive for measuring intermediate levels of virus inactivation observed for some animal viruses. None of these viruses were plaque purified or obtained by reverse genetics. Thus, a broad TCID50 inactivation curve may arise due to heterogeneity in viral sequence. All reverse-genetics derived viruses studied had sharp drops in TCID50 infectivity as a function of pH including rg-A/TN/1-560/2009 (H1N1, and its mutants HA1-Y17H, HA1-Y17H/HA2-V55I, HA2-R106K), rg-A/swine/NC/18161/2002 (H1N1), rg-A/swine/NC/18161/2002 HA1-Y17H (H1N1), rg-A/canine/Indiana/1177-17-1/2017 (PR8 internal genes) (H3N2), and rg-A/canine/Korea/CY009/2010 (PR8 internal genes) (H3N2). Overall, inactivation pH values determined by luciferase assay and TCID50 correlated with an *R*^2^ value of 0.604 (Fig 4).

**Table 1:** Viruses characterized and associated stability values.

#	Virus name^a^	Host	HA Activation pH syncytia^b^	Virus Inact. pH Luc^c^	Virus Inact. pH TCID^d^	Δ pH Activ. minus Inact.^e^	90% Rt at 55°C (min)^f^	Reference^g^
1	rg-A/TN/1-560/2009 HA1-Y17H (H1N1)	human	6.00	5.99	5.91	0.01	5.2 ±1.3	PMID 26811446, 28282440
2	rg-A/TN/1-560/2009 HA1-Y17H/HA2-V55I (H1N1)	human	5.70	5.57	5.57	0.13	8.2 ±1.7	PMID 28282440
3	rg-A/TN/1-560/2009 (H1N1)	human	5.53	5.49	5.41	0.04	14.6 ±2.4	NCBI 646491 (PMID 26811446,28282440)
4	rg-A/TN/1-560/2009 HA2-V55I (H1N1)	human	5.30	5.3		0.00	21.7 ±3.6	PMID 28282440
5	rg-A/TN/1-560/2009 HA2-R106K (H1N1)	human	5.37	5.30	5.23	0.07		PMID 26811446, 28282440
6	rg-A/swine/NC/18161/2002 HA1-Y17H (H1N1)	swine	6.30	6.18	6.09	0.13		NCBI 1081398
7	rg-A/swine/NC/18161/2002 (H1N1)	swine	5.57	5.59	5.54	-0.02		NCBI 1081398
8	A/Bethesda/55/2015 (H3N2)	human	5.50	5.55	5.40	-0.05		PMID 32287326
9	A/Aichi/2/1968 (H3N2)	human	5.25	5.23	5.02	0.02		ATCC VR-547
10	A/Puerto Rico/8/1934 (H1N1)	human	5.25	5.19	4.98	0.06		ATCC VR-1469
11	A/canine/Illinois/41915/2015 (H3N2)	canine	5.50	5.20	4.95	0.30		PMID 32287326
12	rg-A/canine/Indiana/1177-17-1/2017 (PR8 internal genes) (H3N2)	canine	5.40	4.90	4.68	0.50		PMID 32287326
13	A/Ohio/3559/1988 (H1N1)	human	5.60	5.47		0.13		NCBI 416743
14	A/Florida/3/2006 (H1N1)	human	5.30	5.20		0.10		ATCC VR-1893
15	rg-A/California/04/09 (H1N1)	human	5.50	5.40		0.11		NCBI 641501
16	A/TN/F1080/2010 (H1N1)	human	5.40	5.42		-0.02		NCBI 1452594
17	A/TN/F1052d0/2010 (H1N1)	human	5.40	5.36		0.04		NCBI 26811446
18	A/TN/D1076d0/2011 (H1N1)	human	5.35	5.40		-0.05		NCBI 26811446
19	X31, A/Hong Kong/1/1968 (PR8 internal genes) (H3N2)	human	5.30	5.10		0.20		NCBI 132504
20	A/Hong Kong/8/1968 (H3N2)	human	5.30	5.20		0.10		ATCC VR-544
21	A/Victoria/3/1975 (H3N2)	human	5.10	4.99		0.11		NCBI 392809
22	A/Wisconsin/67/2005 (H3N2)	human	5.50	5.39		0.11		ATCC VR-1881
23	A/swine/Wisconsin/1/1961 (H1N1)	swine	5.50	5.57		-0.07	38.2 ±7.2	NCBI 383533
24	A/swine/Wisconsin/30747/1976 (H1N1)	swine	5.70	5.81		-0.11		NCBI 458023
25	A/swine/Minnesota/27/1976 (H1N1)	swine	5.70	5.65		0.05	25.0 ±5.0	NCBI 441574
26	A/swine/TN/84/1977 (H1N1)	swine	5.60	5.67		-0.07		NCBI 441561
27	A/swine/Arizona/148/1977 (H1N1)	swine	5.60	5.65		-0.05		NCBI 441593
28	A/swine/Wisconsin/11/1980 (H1N1)	swine	5.60	5.20	4.65	0.40	15.5 ±4.8	NCBI 441586
29	A/swine/Germany/2/1981 (H1N1)	swine	6.00	5.66		0.34		NCBI 384486
30	A/swine/Maryland/23239/1991 (H1N1)	swine	5.60	5.68		-0.08		NCBI 441597
31	A/swine/California/T9001707/1991 (H1N1)	swine	5.70	5.33	4.78	0.37	21.1 ±1.9	NCBI 457997
32	A/swine/Italy/1369-7/1994 (H1N1)	swine	5.90	5.61	5.45	0.29	23.9 ±3.5	NCBI 1081399
33	A/swine/North Carolina/47834/2000 (H1N1)	swine	5.50	5.49		0.01		NCBI 1081397
34	A/swine/North Carolina/18161/2002 (H1N1)	swine	5.60	5.52		0.08		NCBI 1081398
35	A/swine/Nebraska/4G-0314-P24/2014 (H1N1)	swine	5.30	5.21		0.09		PMID 28282440
36	A/swine/Georgia/1E-0214-P26/2014 (H1N1)	swine	5.20	5.10		0.10		PMID 28282440
37	A/swine/Nebraska/4G-0314-P18/2014 (H1N1)	swine	5.20	5.10		0.10	32.9 ±14.6	PMID 28282440
38	A/swine/Ohio/3987/2010 (H3N2)	swine	5.85	5.69		0.16	12.5 ±2.3	PMID 28282440
39	A/swine/Ohio/3809-2/2010 (H3N2)	swine	5.60	5.47		0.13		PMID 28282440
40	A/swine/Minnesota/4028/2010 (H3N2)	swine	5.50	5.37		0.13		PMID 28282440
41	A/swine/Iowa/2856/2010 (H3N2)	swine	5.50	5.32		0.18		PMID 28282440
42	A/swine/Minnesota/4157/2010 (H3N2)	swine	5.40	5.22		0.18	25.8 ±3.0	PMID 28282440
43	A/swine/Texas/0189/2011 (H3N2)	swine	5.70	5.70		0.00		PMID 28282440
44	A/swine/Minnesota/3908-2/2011 (H3N2)	swine	5.70	5.72		-0.02		PMID 28282440
45	A/swine/Iowa/2514-3/2011 (H3N2)	swine	5.80	5.67		0.14		PMID 28282440
46	A/swine/North Carolina/0033/2011 (H3N2)	swine	5.80	5.53	5.57	0.27	22.5 ±4.3	PMID 28282440
47	A/swine/Iowa/2514-1/2011 (H3N2)	swine	5.60	5.52		0.08		PMID 28282440
48	A/swine/Minnesota/3067/2011 (H3N2)	swine	5.60	5.47		0.13		PMID 28282440
49	A/swine/Arizona/0934/2011 (H3N2)	swine	5.50	5.44		0.06	53.3 ±16.3	PMID 28282440
50	A/swine/Oklahoma/2758/2011 (H3N2)	swine	5.60	5.40		0.20	26.7 ±5.8	PMID 28282440
51	A/swine/North Carolina/0668/2011 (H3N2)	swine	5.50	5.40		0.10		PMID 28282440
52	A/swine/Indiana/0392/2011 (H3N2)	swine	5.50	5.31		0.19		PMID 28282440
53	A/swine/North Carolina/1256/2011 (H3N2)	swine	5.80	5.37	5.57	0.43	16.4 ±6.1	PMID 28282440
54	A/duck/Alberta/35/1976 (H1N1)	duck	5.50	5.30		0.20		BEI NR-21649 (NCBI 691471)
55	A/ruddy turnstone/NJ/65/1985 (H7N3)	ruddy turnstone	5.60	5.14	5.14	0.46		NCBI 279395
56	A/turkey/Ontario/6118/1968 (H8N4)	turkey	5.60	5.42		0.18		BEI NR-21658 (NCBI 311175)
57	A/mallard/Alberta/17/1991 (H9N2)	mallard	5.60	5.49		0.11		NCBI 352598
58	A/duck/England/1956 (H11N6)	duck	5.60	5.37		0.23		BEI NR-21660 (NCBI 383550)
59	A/duck/Alberta/60/1976 (H12N5	duck	5.30	5.22		0.08		BEI NR-43018 (NCBI 385582)
60	rg-A/canine/Korea/CY009/2010 (PR8 internal genes) (H3N2)	canine	5.50	5.13	5.31	0.37		PMID 32287326
61	rg-A/bat/Egypt/381OP/2017 (PR8 internal genes) (H9N2)	bat	6.00	5.33		0.67		PMID 30381492
62	A/swine/Ontario/2/1981 (H1N1)	swine	5.70	5.43	5.53	0.27		SRX6191306, SRP208130
63	A/mallard/Alberta/119/1998 (H1N1)	avian	5.65	5.44	4.82	0.21		PMID 26251829
64	A/pintail/Alberta/210/2002 (H1N1)	avian	5.60	5.30	4.75	0.30		PMID 26251829
65	A/Ruddy Turnstone/Delaware/428/2009 (H1N1)	avian	5.70	5.39		0.31		
66	A/Ruddy Turnstone/Delaware/324/2009 (H1N1)	avian	5.50	5.35	5.05	0.15		
67	A/Ruddy Turnstone/Delaware/274/2009 (H1N1)	avian	5.50	5.57	5.01	-0.07		CY137879
68	A/Ruddy Turnstone/DE/244/1991 (H5N2)	avian	6.20	5.47	5.37	0.73		PMID 8184538
69	A/chicken/Germany/N/1949 (H10N7)	avian	5.45	5.70		-0.25		PMID 32269119
70	A/black-headed gull/Sweden/5/1999 (H16N3)	avian	5.50	5.20	5.33	0.30		PMID 32269119
72	A/mallard/Astrakhan/263/1982 (H14N5)	avian	5.55	5.12	4.56	0.43		PMID 32269119
72	A/duck/Australia/341/1983 (H15N8)	avian	6.00	5.57	5.54	0.43		PMID 32269119
73	A/gull/Maryland/704/1977 (H13N6)	avian	5.60	5.17	5.15	0.43		PMID 32321814

^a^Reverse-genetics-derived viruses denoted with “rg” prefix.

^b^HA activation pH is reported as the highest pH at which virus-infected cells are induced to fuse in a syncytia-formation assay.

^c^Virus inactivation pH measured by luciferase reporter assay.

^d^Virus inactivation pH measured by TCID50 assay.

^e^Difference between HA activation pH and virus inactivation pH measured by luciferase assay.

^f^90% Reduction time expressed in minutes when viruses are incubated at 55°C.

^g^References for viruses include available Genbank identifier (NCBI), Pubmed ID (PMID), ATCC number (ATCC), and BEI Resources ID (BEI).

### Relationship between HA protein activation pH and virion inactivation pH

**Fig 5:**
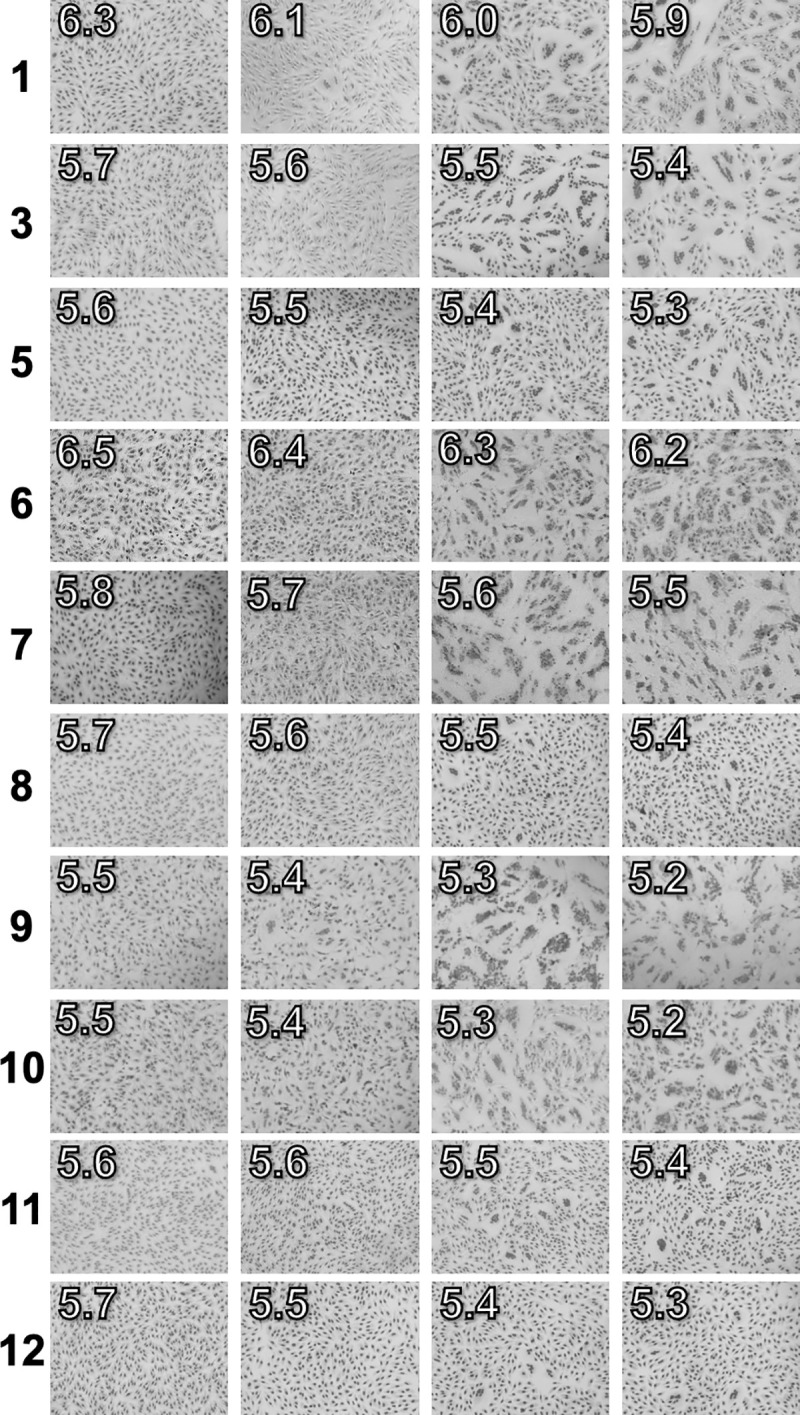
HA activation pH values measured by syncytia formation assay. Viruses identified by the numbers on the left side of each row (*cf*. Table 1) were inoculated into Vero cells at an MOI of 3 PFU/cell. The pH values for PBS overlaid onto the cells are reported on the top left of each microscopic image. HA activation pH was defined as the highest pH at which syncytia formation occurred (third column).

We next measured HA activation pH, or the pH at which the HA protein is triggered to cause membrane fusion, by syncytia formation assays using virus-infected Vero cells. HA activation pH values for A/TN/09 WT, Y17H, and R106K were 5.5, 6.0, and 5.4, respectively (Fig 5), similar to their virus inactivation pH values (Table 1). HA activation and virus inactivation pH values were also similar for rg-A/swine/NC/18161/2002 (H1N1) HA WT, rg-A/swine/NC/18161/2002 (H1N1) HA1-Y17H, A/Bethesda/55/2015 (H3N2), A/Aichi/2/1968 (H3N2), and A/Puerto Rico/8/1934 (H1N1). In contrast, A/canine/Illinois/41915/2015 (H3N2) and rg-A/canine/Indiana/1177-17-1/2017 (H3N2) HA/NA (PR8 internal genes) had HA activation pH values at least 0.4 pH-units higher than their virus inactivation pH values (Table 1 and Fig 5). This discrepancy suggested HA activation pH and virion inactivation pH may not be equivalent for all IAV isolates.

**Fig 6:**
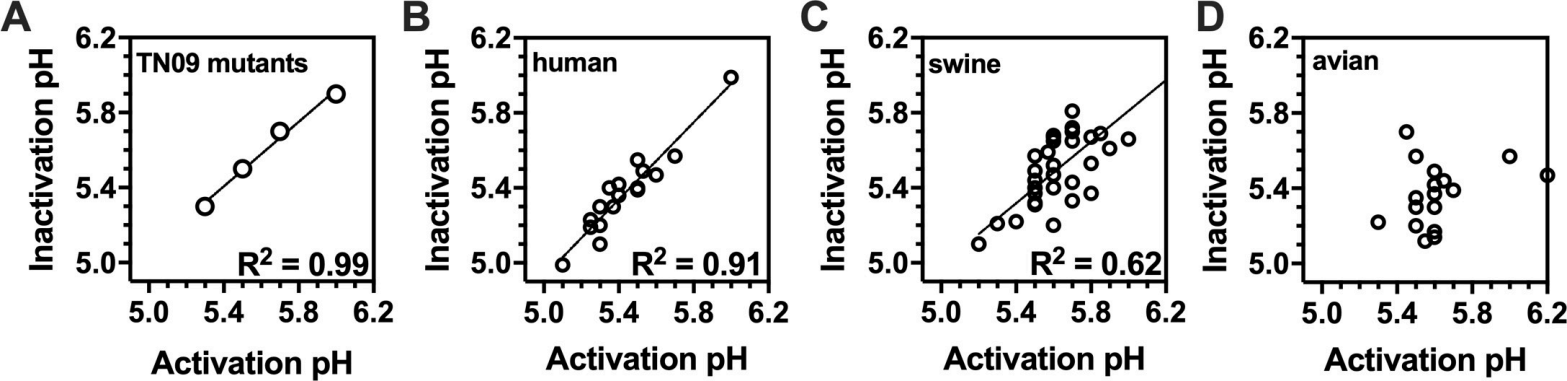
Comparison of HA activation pH and virion inactivation pH values. HA activation pH values were measured by syncytia assay, and virion inactivation pH values were measured by luciferase reporter assay. (A) Comparison for A/TN/09 pH1N1 variants Y17H, Y17H/V55I, WT, and V55I. (B) Comparison for 18 human IAVs. (C) Comparison for 34 swine IAVs. (D) Comparison for 17 avian IAVs. Values shown are average values for at least two independent experiments. The data was analyzed by simple linear regression using GraphPad Prism 8 and calculated R^2^ values are reported in the bottom-right corners of panels A-C.

Altogether, we measured HA activation pH and virus inactivation pH for four A/TN/09 HA variants, 18 human H1N1 and H3N2 viruses, 34 swine H1N1 and H3N2 viruses, 17 avian isolates of varying subtype, 3 canine H3N2 viruses, and a recent bat H9N2 isolate (Table 1). For the A/TN/09 HA variants, HA activation pH and virus inactivation pH correlated with an *R*^*2*^ value of 0.99 (Fig 6A). HA activation pH and virus inactivation pH values also correlated highly (*R*^*2*^ = 0.91) for the 18 human viruses from 1968–2011 (Fig 6B). For the 34 swine viruses from 1961–2014, an increase in HA activation pH correlated with an increase in virus inactivation pH (Fig 6C), albeit to a lesser extent (*R*^*2*^ = 0.62) than for the human viruses. Three swine viruses had HA activation pH values 0.4 units greater than virion inactivation pH: A/swine/Wisconsin/11/1980 (H1N1), A/swine/California/T9001707/1991 (H1N1), and A/swine/North Carolina/1256/2011 (H3N2). Substantial differences between HA activation pH and virus inactivation pH were also observed for the avian viruses (Fig 6D), the bat virus, and the canine viruses (Table 1).

**Fig 7:**
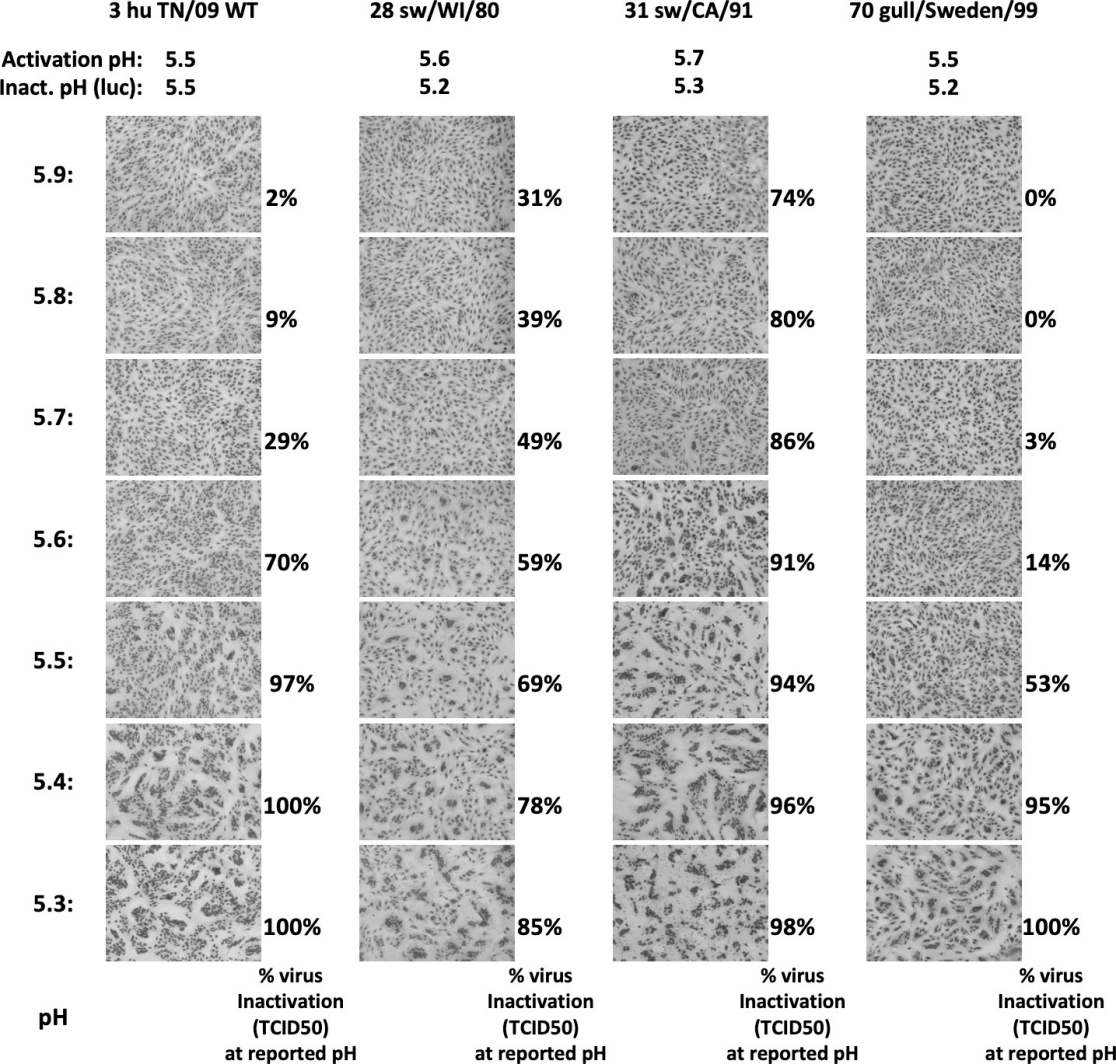
Comparison of percent virus inactivation and syncytia formation as a function of incubation pH. The percent of virus inactivation by TCID50 assay was calculated at pH values ranging from 5.3 to 5.9 at 0.1-unit increments. These values are displayed to the right of associated micrographs. HA activation pH values by syncytia assay and inactivation pH values by luciferase assay are provided at the top of each column for reference. The viruses are A/TN/09 WT, A/swine/Wisconsin/11/1980 (H1N1), A/swine/California/T9001707/1991 (H1N1), and A/black-headed gull/Sweden/5/1999 (H16N2).

We investigated further the shift in virus inactivation pH compared to HA activation pH using two swine viruses and one avian virus. Using TCID50 data, we calculated the % virus infectivity remaining after exposure to pH media that ranged from 5.3 to 5.9, and we displayed these values next to syncytial micrographs (Fig 7). For A/swine/Wisconsin/11/1980 (H1N1), A/swine/California/T9001707/1991 (H1N1), and A/black-headed gull/Sweden/5/1999 (H16N2), the % virus inactivation at the highest pH at which syncytia formation occurred was 59%, 86%, and 53%, respectively. Exposure to media that caused >90% virus inactivation was associated with more extensive syncytia formation. For A/TN/09 WT, a virus with identical HA activation pH and virus inactivation pH values, exposure to pH 5.6 media caused 70% virus inactivation but was insufficient to induce syncytia formation. Syncytia were first observed for A/TN/09 WT at pH 5.5, a value that inactivated 97% of virions. Like the other human viruses studied here, A/TN/09 WT had a sharp acid inactivation curve and required acidification to a relatively high level before its HA protein was triggered to cause membrane fusion. In contrast, the three animal viruses had broader acid inactivation curves and were able to induce membrane fusion at lower levels of acidification relative to virus inactivation.

### Relationship between thermal and acid stability of influenza virions

**Fig 8:**
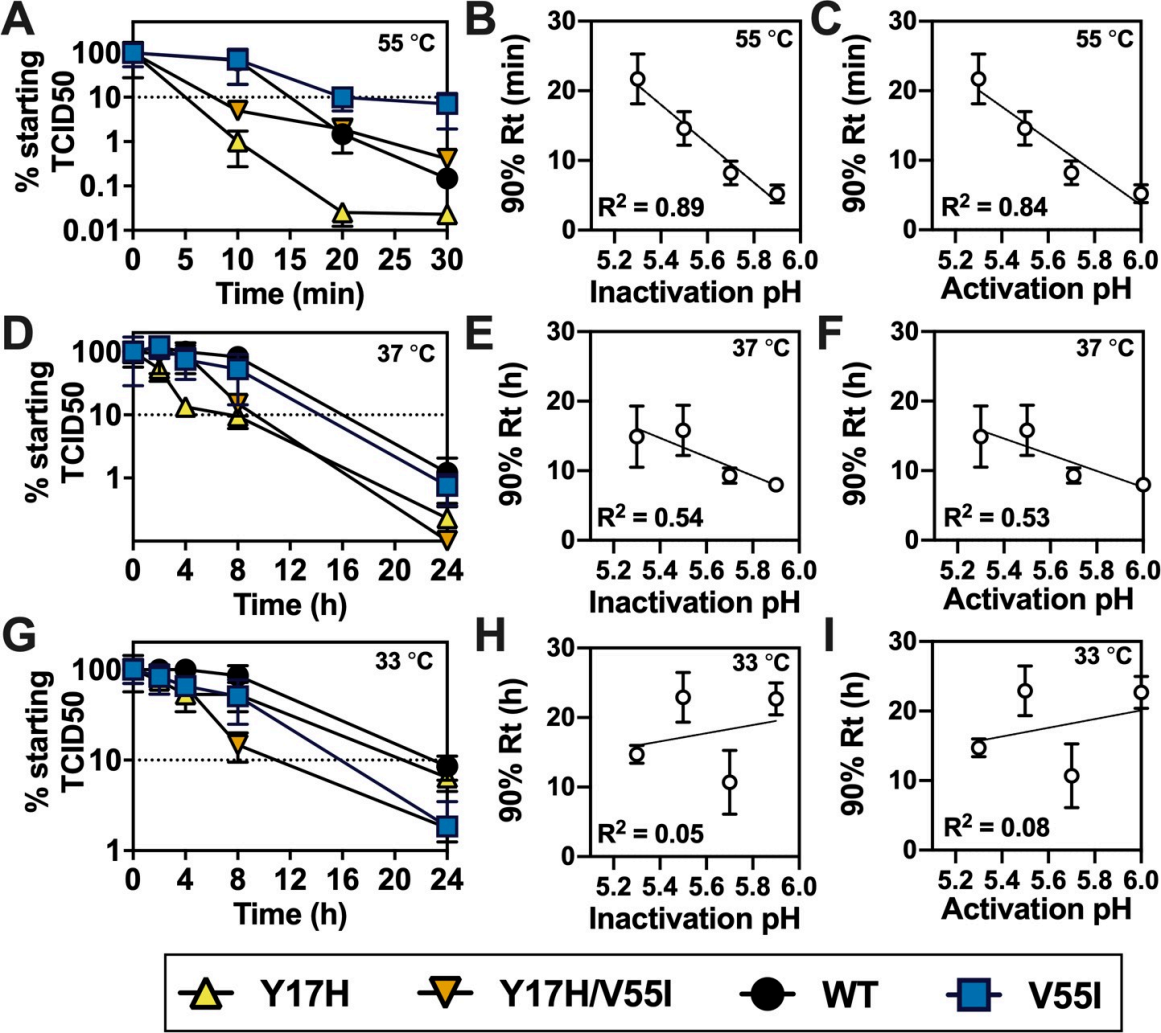
Virus thermostability of A/TN/09 variants and its relationship to pH stability. Residual infectivity (normalized TCID50) was determined for virus-containing aliquots incubated at pH 7.0 for the reported times at 55°C (A), 37°C (D), and 33°C (G). Viruses are Y17H (yellow triangles), Y17H/V55I (orange triangles), WT (black circles), and V55I (blue squares). Virus numbers are 1–4. The 10% (dotted) line corresponds to a 90% reduction of starting infectivity. Virion inactivation pH and HA activation pH values are compared to calculated 90% Reduction time (Rt) values measured at 55°C (B-C), 37°C (E-F), and 33°C (H-I). Representative data from a repeated experiment is shown. Data were analyzed by simple linear regression using GraphPad Prism 8 and calculated R^2^ values are reported in the bottom-left corners.

It has become common to probe HA stability by measuring residual viability as a function of incubation time at elevated temperature (usually in the range of 50–60°C and most often at 55°C) by HA assay [25, 27, 36, 57, 74–76], TCID50 [57], or plaque assay [27, 74, 76, 77]. However, the relationship between HA stability and virus thermal stability has not been thoroughly investigated. In the present study, we incubated aliquots of A/TN/09 variants (WT, Y17H, V55I, and Y17H/V55I) at 55°C and measured residual infectivity as a function of time. Despite requiring more time and assay plates than the luciferase assay, TCID50 was used to measure residual infectivity to align better with previous studies. The destabilizing mutation Y17H induced quicker virus inactivation at 55°C while the stabilizing mutation V55I promoted longer infectivity compared to A/TN/09 WT (Fig 8A). The 90% reduction time (Rt value), or the time required for a 90% (or 1 log10) decrease in the viral titer [38], for the A/TN/09 variants correlated inversely with both virus inactivation pH and HA activation pH (*R*^*2*^ values of 0.89 and 0.84, respectively) (Fig 8B and 8C). Thus, for the variants that shared a common genetic backbone, virion stability at the supraphysiological temperature of 55°C accurately reflected the acid stability of the HA protein and influenza virions.

Virus thermostability at 37 and 33°C reflects the ability of extracellular virions to avoid inactivation at physiologically temperature and neutral pH. A virus with decreased thermostability at physiological temperature is expected to be attenuated due to increased extracellular inactivation [73]. Thermal stability was a relatively poor predictor of acid stability of the A/TN/09 variants at physiological temperatures of 37 and 33°C (Fig 8D–8I). For example, the V55I virus had a 90% Rt value similar to WT at 37°C despite V55I having HA activation pH and virus inactivation pH values 0.2 units lower than WT (Fig 8E and 8F). The viruses Y17H/V55I to Y17H also had similar 90% Rt values while differing in acid stability by 0.2–0.3 units. At 33°C, 90% Rt values did not correlate with virus inactivation pH and HA activation pH (*R*^*2*^ = 0.06 and 0.08, respectively) (Fig 8H and 8I). In summary for the four A/TN/09 variants, thermal stability at the supraphysiological temperature of 55°C correlated with acid stability but thermal stability at the physiologically relevant temperatures of 33 and 37°C did not.

**Fig 9:**
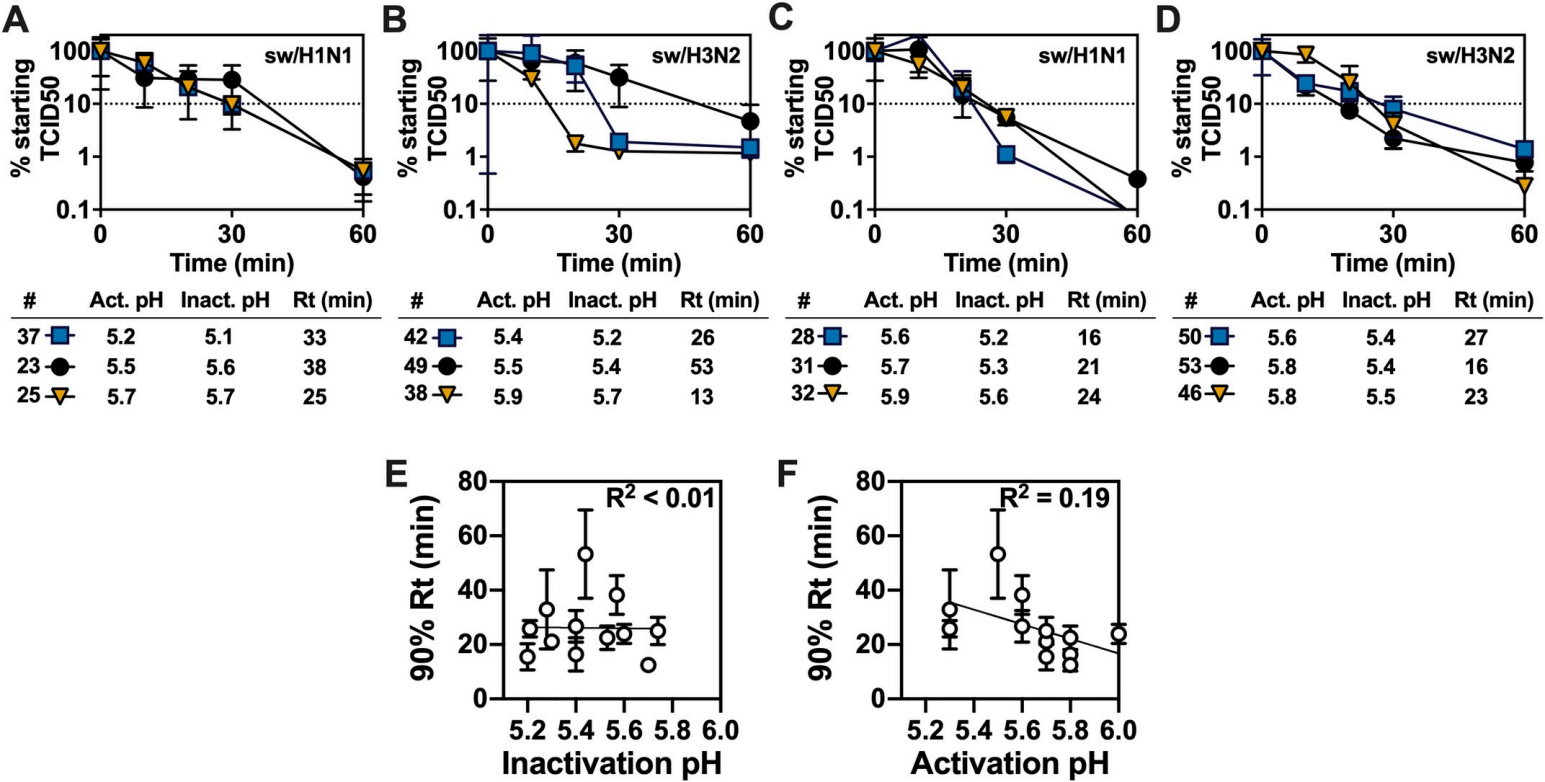
Virus thermostability of swine H1N1 and H3N2 isolates and its relationship to pH stability. (A-D) Residual infectivity (normalized TCID50) determined for virus-containing aliquots incubated at pH 7.0 for the reported times at 55°C. Virus numbers and their associated symbols, HA activation pH, inactivation pH, and Rt values are listed below each panel. The 10% (dotted) line corresponds to a 90% reduction of starting infectivity. (E-F) Virion inactivation pH and HA activation pH values compared to calculated 90% Reduction time (Rt) values measured at 55°C. Representative data from a repeated experiment is shown. Data were analyzed by simple linear regression using GraphPad Prism 8 and calculated R^2^ values are reported in the top-right corners.

To examine the relationship between thermal and acid stability for IAVs that differ genetically, thermal inactivation and associated 90% Rt values at 55°C were measured for twelve swine IAV isolates (Fig 9). For these genetically distinct swine viruses, 90% Rt values at 55°C did not correlate with virus inactivation pH (Fig 9E, *R*^*2*^ < 0.01) and HA activation pH (Fig 9F, *R*^*2*^ = 0.19). Fig 9 groups the viruses into triplets for ease of analysis: (A) H1N1 with similar activation and inactivation pH values, (B) H3N2 with similar activation and inactivation pH values, (C) H1N1 with HA activation higher than virus inactivation pH, and (D) H3N2 with HA activation higher than virus inactivation pH. Triplet A contained swine H1N1 viruses A/swine/Nebraska/4G-0314-P18/2014 (virus #37, activation pH 5.2, inactivation pH 5.1), A/swine/Wisconsin/1/1961 (#23, 5.5, 5.6), and A/swine/Minnesota/27/1976 (#25, 5.7, 5.7). In this group, viruses 25 and 37 had similar thermal inactivation kinetics but differed by approximately 0.5 pH units in pH stability (Fig 9A). Triplet B contained swine H3N2 viruses A/swine/Minnesota/4157/2010 (#42, 5.4, 5.2), A/swine/Arizona/0934/2011 (#49, 5.5, 5.4), and A/swine/Ohio/3987/2010 (#38, 5.9, 5.7). The least acid-stable virus (#38) was inactivated the quickest at 55°C as might be expected; however, virus #42 was more acid stable than virus 49 but was shorter lived at 55°C (Fig 9B). Triplets C and D contained swine IAVs that had HA activation pH values 0.2–0.4 units higher than virion inactivation pH. Triplet C contained swine H1N1 viruses A/swine/Wisconsin/11/1980 (#28, 5.6, 5.2), A/swine/California/T9001707/1991 (#31, 5.7, 5.3), and A/swine/Italy/1369-7/1994 (#32, 5.9, 5.6). The most acid-stable virus (#28) was shorter lived at 55°C than the other two (Fig 9C). Triplet D contained A/swine/Oklahoma/2758/2011 (#50, 5.6, 5.4), A/swine/North Carolina/1256/2011 (#53, 5.8, 5.4), and A/swine/North Carolina/0033/2011 (#46, 5.8, 5.3). Viruses 46 and 53 had similar pH stability, but virus 46 was inactivated substantially slower at 55°C than virus 53 (Fig 9D). In summary, thermal stability at 55°C did not correlate with acid stability for the twelve swine H1N1 and H3N2 isolates.

## Discussion

In this work, we developed a reporter assay using MDCK-Luc9.1 cells to measure the pH of IAV inactivation, and we used this and other assays to investigate the relationships between HA activation pH (HA stability), virion inactivation pH (IAV acid stability), and virion thermostability. Thermostability of viruses containing HA point mutations correlated with HA stability at elevated temperature (55°C) but not at physiological temperatures (33 and 37°C). This suggests the thermostability assay should only be used to compare HA stabilities of mutant viruses incubated at supraphysiologic temperature. At 37°C, HA activation pH was at least 0.2 units higher than virus inactivation pH for one-quarter of the swine viruses, two-thirds of the avian viruses, all 3 canine viruses, and the single bat virus studied. Both a relatively broad acid inactivation curve and more facile triggering of membrane fusion appeared to contribute to HA activation pH being higher than virus inactivation pH for the animal viruses. The average body temperatures of swine and many avian species are approximately three degrees higher than that of humans; therefore, animal-adapted IAVs may benefit from greater virion stability relative to their HA stabilities. Uncorrelated HA activation pH and virion inactivation pH values have also been reported in the literature for A/chicken/C58/2004 (H5N1), several canine H3N2 viruses, A/turkey/Ontario/6118/68 (H8N4), and A/duck/England/56 (H11N6) [25, 26, 70, 71]. For all the human IAVs studied here, HA activation pH correlated with virus inactivation pH. The results suggest that human-adapted viruses may require HA acid stability to closely match virus acid stability, while animal-adapted viruses may be more tolerant of a shift between the two properties.

While HA activation pH values of human-adapted viruses tend to range from pH 5.0–5.5, those from IAVs isolated from swine, avian, and other species have a broader range [51]. A relatively stable HA protein, with an activation pH of approximately 5.5 or less, has been linked to both the adaptation of 2009 pH1N1 to humans [34, 35, 37, 78] and the airborne transmissibility of H1N1, H3N2, and H5N1 viruses in ferrets [27, 28, 31–34, 41, 79, 80]. The present study showed that human-adapted IAVs have similar HA activation pH and virion inactivation pH values. This may be due because human IAV transmission requires (a) extracellular virions avoid inactivation in the mildly acidic upper respiratory tract and while transmitting between hosts in airborne particles [41, 73, 81] and (b) endosomal entry that minimizes innate antiviral responses triggered by HA proteins that are too stable or unstable [45, 73].

In contrast to human IAVs, some of the animal IAVs had an HA activation pH higher than the respective virus inactivation pH. Overall, swine IAVs have a broader range of HA activation than human IAVs [51], ranging from pH 5.0–6.3 in the present study and others [32, 33, 79, 82]. Individual clades of swine viruses may trend toward higher or lower HA stability. For example, H1N1 gamma clade viruses isolated from 2012 to 2016 have HA activation pH values ranging from 5.5–5.9 and H1N1 pandemic clade viruses isolated from 2009 to 2016 range from 5.0–5.5 [32]. Compared to ferrets, swine tolerate a broader range of HA activation pH for efficient replication and transmission, suggesting swine may serve as an intermediate host for the adaptation human-like HA stability [33]. Two-thirds of the avian isolates studied here have substantial differences between HA activation pH and virus inactivation pH, but the human-adapted isolates did not. The fact that one-quarter of the swine isolates had a mismatch between the two properties suggest swine might serve as an intermediate host to refine HA stability and virion stability to a human-preferred level just as swine serve as an intermediate host for the adaptation of human-preferred receptor-binding specificity.

Despite shifts in virion stability compared to HA stability for some animal isolates, the data reported here show that HA acid stability is a primary driver of IAV inactivation after exposure of virions to extracellular low pH. Fusion of the viral envelope and endosomal membrane during viral entry is mediated by acid-induced activation of the HA protein and results in irreversible HA conformational changes [24]. Activation of the HA protein from its metastable prefusion form is biologically triggered by low pH at physiological temperatures but can be artificially induced by exposure to mild denaturant or supraphysiological heating at neutral pH [21–23]. Regardless of the triggering force, activation of all HA trimers in extracellular virions would lead to IAV inactivation upon subsequent receptor binding and internalization. For 2009 human pandemic and swine gamma-lineage H1N1 viruses containing HA stalk mutations, pH values of membrane fusion and virion inactivation have been reported to be similar (within 0.15 pH units) [32, 34, 44]. Correlations between virion inactivation pH [25] and HA activation pH [26] measured in different studies have also been observed for A/Hong Kong/1/68 (H3N2), A/duck/Ukraine/63 (H3N8), and A/chicken/Germany/N/49 (H10N7). A similar correlation was noted in the present study for 14 human IAVs of H1N1 and H3N2 subtypes isolated between 1934 and 2011. Because HA acid stability is a key driver of virion longevity and infectivity, IAV strains with suboptimal HA stability may need to be engineered with stability-altering HA mutations to enhance vaccine production, infectivity, and immunogenicity for live attenuated viruses [36, 83, 84].

The significance of an apparent decoupling of HA activation and virion inactivation pH for some IAV isolates is that false equivocation of these two properties could lead to a misunderstanding of the necessary property (HA stability versus virion resistance to acid inactivation) for IAV host range, adaptation to humans, transmissibility, and pathogenicity. Based on studying viruses with similar HA activation and virion inactivation pH values, a value lower than 5.6 has been considered necessary for adaptation to humans and ferrets [27–34]. However, it is currently unknown whether an HA activation pH, virion inactivation pH, or both lower than 5.6 is required. The present study suggests that HA stability and virion stability may both need to be similarly stable for human adaptation. Not understanding which property needs to be less than pH 5.6 for human adaptation could lead to false negatives or positives when using the wrong assay for risk assessment algorithms to screen IAV surveillance isolates. For example, it is unclear if A/swine/California/T9001707/1991 (H1N1), which has an HA activation pH of 5.7 and a virion inactivation pH of 5.3, has the proper form of stability required for human adaptation. Overall, the discovery of a decoupling of HA and virion stability in some isolates means risk assessment studies should include both types of assays until mechanistic studies are performed to establish which trait, or both, is needed for influenza pandemic risk.

Several mechanisms may cause a shift between HA activation pH and virus inactivation pH. In the present work, many of the viruses with activation/inactivation mismatches had relatively broad acid-dependent inactivation curves in the TCID50 assay. This suggests that at an upper threshold pH, some cell-surface expressed HA trimers are triggered to cause membrane fusion while other HA trimers in virions remain in a fusion-capable form and avoid full virion inactivation. Mutations in the M and NA genes have been shown to modulate HA stability and virus entry [57, 68, 80, 85, 86]. Thus, functional interactions between the HA protein and the NA, M, and M2 proteins may cause a shift in virus inactivation pH compared to activation pH. Viral genetic heterogeneity in a sample could allow a relatively unstable HA subpopulation to become activated at a given pH while a more-stable subpopulation resists inactivation. Moreover, it is possible that morphological heterogeneity between virions could allow a subset of virions to be capable of membrane fusion at a given pH while a morphologically distinct, yet genetically identical, subset is able to resist inactivation.

An inverse correlation between the pH and temperature of membrane fusion for IAV viruses containing HA protein mutations was first shown for X-31 [87]. Based on this and the related observation that membrane fusion can be triggered artificially by heating [60], surrogate assays for HA stability have included measurements of virion infectivity or HA activity as a function of incubation time at supraphysiological temperature (usually 50–60°C) [27, 36, 57, 74–77] or the temperature required to inactivate virions after 30 minutes exposure at neutral pH [31, 36, 88]. Despite recent usage in the field, thermal inactivation assays have several noteworthy limitations. First, absolute values for thermal stability have not been correlated with biological properties of IAV strains; therefore, such assays may not provide data helpful in screening isolates for risk-assessment algorithms. Second, the present study showed that the rate of inactivation of pH1N1 viruses containing HA mutations at the supraphysiological temperature of 55°C did not correlate with inactivation at biologically relevant temperatures of 33 and 37°C. This suggests virion inactivation during supraphysiological heating is a passenger property of viruses and not a biological driver. Most importantly, heat stability at 55°C correlated with acid stability for isogenic HA mutants but not for genetically distinct H1N1 and H3N2 swine viruses. Therefore, factors other than HA stability and/or virion acid stability appear to modulate stability at supraphysiological temperatures; these unknown factors are most likely not operational under physiological conditions because the effects are lost during incubation at 33 and 37°C.

In conclusion, this work showed that HA stability is a major, but not exclusive, determinant of IAV persistence after exposure to low pH, and it described a new luciferase-reporter assay for more efficient measurement of IAV inactivation as a function of pH. The observation that IAVs have similar HA activation pH and virus inactivation pH values, while many swine and avian viruses do not, suggests that human-adapted IAVs may require a coupling of the two properties. This suggests influenza risk assessment algorithms may be enhanced by adding measurements of HA and virion stability in addition to receptor-binding specificity.

## Materials and methods

### Cell lines

Madin-Darby canine kidney (MDCK; ATCC CCL-34) cells were cultured and maintained in Dulbecco’s modified Eagle’s medium (DMEM) supplemented with 5% fetal bovine serum (FBS) and 1% penicillin-streptomycin (Pen-Strep). Vero cells (ATCC CCL-81) were maintained in DMEM supplemented with 10% FBS and 1% Pen-Strep. MDCK-derived Luc9.1 reporter cells (RRID:CVCL_GY95) developed by Hossain, Guo, and Donis [72] were incubated in DMEM supplemented with 10% FBS, 1% Pen-Strep, and 500μg/mL G418 (Geneticin, every other passage). All cells were maintained in a 5% CO2 incubator at 37°C.

### Viruses and virus propagation

All viruses used in this study are listed in Table 1, which also lists ATCC, BEI Resources, NCBI, and PMID references when available. Virus titer was measured by plaque assay, and viruses were amplified one round in Madin-Darby Canine Kidney (MDCK) cells if the titer was low (<1x10^4^ PFU/ml). Briefly, MDCK cells were seeded in T25 or T75 tissue culture flasks the day before virus infection. Cells were washed twice by PBS buffer, inoculated with virus inoculum (MOI = 0.01–0.001 PFU/cell), and incubated at 37°C, 5% CO_2_ for 1 h. During the incubation, flasks were gently rocked several times every 15 minutes, allowing the inocula to cover the cells. Inocula were then aspirated, and virus growth medium was added to the flask. The growth medium was 1x Minimum Essential Medium (MEM) with a 4% bovine serum albumin (BSA) solution, 1% antibiotics-antimycotic (100x), 1% MEM vitamins solution (100x), 2 mM L-glutamine (200mM), 40mg/L gentamicin sulfate, and 3% sodium bicarbonate (7.5%) solution, and a final concentration of 1μg/ml of TPCK-treated trypsin. After 3 d incubation at 37°C in a 5% CO_2_ incubator, virus was harvested, aliquoted, frozen in a -80°C freezer, and quantified by plaque titration for future use.

### TCID50 assay

Virus samples were serially diluted 10-fold and then loaded onto PBS-washed MDCK cells in 96-well tissue culture plates. After 3 d at 37°C in a 5% CO_2_ incubator, supernatants were transferred to round-bottom 96-well plates so that HA assays could be performed and median Tissue Culture Infectious Dose (TCID50) values could be calculated by standard methods [89].

### pH buffer preparation

pH-adjusted buffers were prepared fresh for each experiment. An AccupHast (Fisher Scientific) pH meter was calibrated each time using a standard buffer solution. 0.1 M citric acid was used to adjust the pH of PBS buffers.

### Syncytia assay to measure HA activation pH

HA activation pH values were determined by syncytia formation assays in Vero cells as previously described [63]. Vero cells were seeded in 6-, 12- or 24-well tissue culture plates 1 d before virus infection. Confluent monolayers of Vero cells were washed with PBS two times and were infected with IAVs at an MOI of 3 PFU/cell for 1 h. After infection, infection medium was aspirated, viral growth medium without TPCK-treated trypsin was overlaid, and culture dishes were incubated at 37°C in a 5% CO_2_ incubator for 17 hours overnight. After incubation, virus growth media was aspirated, and Vero cells were rinsed twice with PBS buffer. Cells were treated with 5 μg/mL TPCK-treated trypsin for 5 min, and then the trypsin was inactivated using DMEM with 5% FBS. Media was aspirated, PBS buffer was used to wash cells twice, and then Vero cells were overlaid with pH-adjusted PBS buffers for 5 min at 37°C. DMEM containing 5% fetal bovine serum was added to the cells, which were incubated an additional 3 h at 37°C. Vero cells were then fixed and stained with a Hema 3 Stat Pack (Fisher Scientific, Kalamazoo, MI) according to the manufacturer’s protocol. Photomicrographs were taken using a Nikon Digital Sight camera affixed to a Nikon Eclipse TS100 light microscope. HA activation pH was reported as the highest pH value at which heterokaryon formation was observed.

### Virus acid and thermal inactivation assays using TCID50 as readout

To measure the abilities of the viruses to retain or lose infectivity as a function of exposure to solutions of varying pH, 5 μl virus stocks (usually 10^5^~10^7^ PFU/ml) were added to 495 μl of pH-adjusted buffer in 1 ml deep-well plates. The samples were mixed well by pipetting up and down. Plates were covered and incubated for 1h at 37°C. After the acid treatment, 90 μl of virus-containing sample was transferred to 810 μl virus infection medium to re-neutralize the samples [71]. For thermal inactivation assays, virus aliquots were added to 500 μl PCR tubes or 1 ml Eppendorf tubes, then placed in a temperature-adjusted thermocycler or water bath until flash cooling at the designated time. TCID50 values were determined as described above.

### Virus acid inactivation assays using luciferase reporter cells

One day before the experiment, 96-well white tissue culture plates (Costar, REF 3917) were seeded with 1x10^6^ MDCK-derived Luc9.1 cells in DMEM growth medium containing 5% FBS and 1% Pen-Strep supplemented. After pH or thermal exposure as described in the section above, 96-well plates containing confluent Luc 9.1 reporter cells were washed twice with PBS buffer before virus samples were loaded (200 μl/well). *Renilla* luciferase enzymatic activity was used to measure the viral infectivity. In brief, about 17–19 hours post virus infection, plate medium was dumped to a waste container with 5% Micro-Chem Plus detergent/disinfectant in the biological safety cabinet, 20 μL *Renilla* luciferase lysis buffer was directly added to each well, plates were placed on ice for 30 minutes with occasionally gently shaking to let the lysis buffer cover the cells in the plate, and plates were read after 100-μl diluted *Renilla* luciferase substrate was added to each well under Veritas luminometer. Susceptibility to inactivation by exposure to low pH was reported as the inflection pH calculated using GraphPad Prism 8 [XY analysis, nonlinear regression (curve fit), log(agonist)vs. response-Variable slope (four parameters)]. Baseline signal threshold for negative control samples was calculated as the mean of the negative control values (relative light units, RLU) plus three times of the standard deviation of those values. 90% reduction time (Rt) values at were calculated by straight-line nonlinear regression between adjacent data points that buttressed a 10-fold reduction in infectivity using GraphPad Prism 8.

## Supporting information

S1 Fig Acid inactivation titrations performed using 0.5 and 0.2 pH-unit steps. Virus inactivation titrations were performed using Luc9.1 cells. Viruses used were A/TN/09 WT (solid circles), HA1-Y17H (open triangles), and HA2-R106K (gray squares) at an MOI 0.2 PFU/cell. Virus aliquots were incubated with pH-adjusted PBS at 0.5-unit steps (A-C) or 0.2-unit steps (D-F). After reneutralization in media supplemented with TPCK-treated trypsin, virus samples were loaded onto Luc9.1 cells and then incubated for 17 h before luminescence was measured as relative light units (RLU). Midpoints of inactivation, or inactivation pH values, are listed on the panels. Dotted lines correspond to the limit of detection (3 standard deviations above the mean) of uninfected negative control samples. Error bars represent standard deviation (n = 3). Reported data are representative of three independent experiments.
(TIF)
