# Supplementary figures and images for: Relationship between hemagglutinin stability and influenza virus persistence after exposure to low pH or supraphysiological heating
Source: PLoS Pathog. 2021 Sep 3;17(9):e1009910. doi: 10.1371/journal.ppat.1009910 (PMC8445419; doi:10.1371/journal.ppat.1009910)

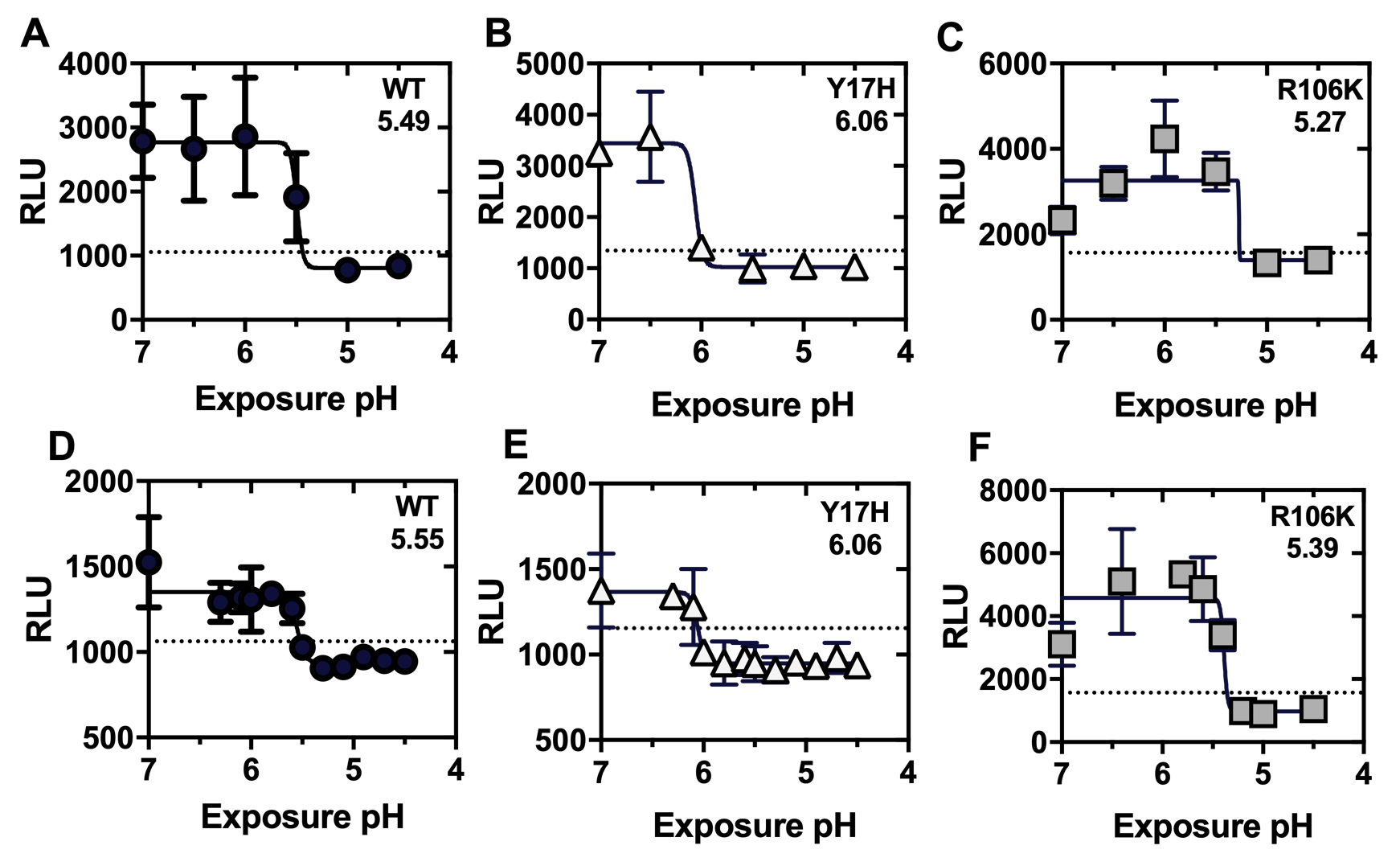

Supplement: S1 Fig — Virus inactivation titrations were performed using Luc9.1 cells. Viruses used were A/TN/09 WT (solid circles), HA1-Y17H (open triangles), and HA2-R106K (gray squares) at an MOI 0.2 PFU/cell. Virus aliquots were incubated with pH-adjusted PBS at 0.5-unit steps (A-C) or 0.2-unit steps (D-F). After reneutralization in media supplemented with TPCK-treated trypsin, virus samples were loaded onto Luc9.1 cells and then incubated for 17 h before luminescence was measured as relative light units (RLU). Midpoints of inactivation, or inactivation pH values, are listed on the panels. Dotted lines correspond to the limit of detection (3 standard deviations above the mean) of uninfected negative control samples. Error bars represent standard deviation (n = 3). Reported data are representative of three independent experiments. (TIF) [file ppat.1009910.s001.tif]
